# Evaluating the Use of Chemical Weapons for Capturing Prey by a Venomous Mammal, the Greater Slow Loris (*Nycticebus coucang*)

**DOI:** 10.3390/ani14101438

**Published:** 2024-05-11

**Authors:** Grace Fuller, Wirdateti, K. A. I. Nekaris

**Affiliations:** 1 Nocturnal Primate Research Group, School of Social Sciences and Law, Oxford Brookes University, Oxford OX3 0BP, UK; anekaris@brookes.ac.uk; 2 Detroit Zoological Society, Royal Oak, MI 48067, USA; 3 Division Zoology, Research Center for Biosystematics and Evolution, Badan Riset dan Inovasi Nasional (BRIN), Kawasan Sains dan Teknologi (KST), Soekarno, Cibinong 16911, Indonesia; teti_mzb@yahoo.com

**Keywords:** venomous mammal, strepsirrhine, tongue-flicking, chemosensory behavior, predator-prey interactions

## Abstract

**Simple Summary:**

Having venom is a rare trait among mammals and even rarer among primates. Slow and pygmy lorises are the only venomous primates, and they possess a unique “two-step” venom system. When threatened, they release a secretion from a gland on their forearm and lick it, activating the venom by mixing it with their saliva. There are several hypotheses for why slow and pygmy lorises evolved this unique trait. Venom can be used to capture prey, to defend against predators or parasites, or for competition with other slow or pygmy lorises. We tested the hypothesis that venom is used to capture prey by experimentally offering various arthropod prey items to 22 wild-caught greater slow lorises living in a rescue center. We observed how their behavior was affected by prey characteristics including size, potential for escape, and toxicity. The few venom-related behaviors we observed only occurred in a defensive context, suggesting that the greater slow lorises do not use their venom as a means of subduing prey. These negative results are consistent with the growing body of evidence that pygmy and slow lorises primarily use venom in competition with members of their own species.

**Abstract:**

Few mammals are venomous, including one group of primates—slow (*Nycticebus* spp.) and pygmy (*Xanthonycticebus* spp.) lorises. Hypotheses for the evolutionary function of venom in these primates include defense from predators or ectoparasites, communication or competition with conspecifics, and the capture of prey. We tested the prey capture hypothesis in 75 trials with 22 wild-caught greater slow lorises (*N. coucang*) housed in a rescue center in Java, Indonesia. We experimentally offered the slow lorises arthropod prey items varying in size, escape potential, and toxicity and recorded venom-related and predatory behaviors using live and video observations. The slow lorises visually targeted arthropod prey, approached it quickly and efficiently, and captured it with a manual grasping motion. They rarely performed venom-related behaviors and seemed to do so in a defensive context. The slow lorises exhibited little variation in pre-capture behavior as a function of prey size or escape potential. In response to noxious prey, the slow lorises performed tongue-flicking and other investigative behaviors that indicate they are using chemosensory input to assess prey characteristics. These data suggest it is unlikely that slow lorises use chemical weapons to subdue arthropod prey and may support, instead, a defensive function for slow loris venom.

## 1. Introduction

The evolution of venom has occurred rarely in mammalian taxa, and slow (*Nycticebus* spp.) and pygmy (*Xanthonycticebus* spp.) lorises (hereafter referred to as slow lorises) are unique among primates in possessing a toxic bite [1,2,3]. When threatened, slow lorises release an exudate from the brachial gland (BGE) that, when mixed with their saliva, is fatal to small prey, such as mice [4], and may cause festering wounds or anaphylactic shock in humans [5]. While there is some debate whether loris exudate constitutes a true ‘venom’, here, we use the broad definition from Fry [6] that defines ‘venom’ as a substance produced by a specialized gland in one animal delivered to another by inflicting a wound. The effect of the substance on the receiver facilitates feeding or defense in the venomous animal [6]. Researchers have suggested that BGE may change over time in animals brought into captivity, with the potential for slow lorises to sequester some compounds involved in BGE from their diets [3]. Dietary sources of BGE components could include arthropods, which are abundant in slow loris habitats [7] and play an important role in their diet [8,9,10,11].

Several hypotheses have been posited for the evolution of venom in slow lorises. A decade of research has revealed that venom is most certainly used in intraspecific competition between lorises [12]. For many taxa, however, venom may have multiple uses and may have evolved for one function initially, diversifying later [13]. In slow lorises, venom has been hypothesized to play potential adaptive roles in anti-predator behavior, intraspecific communication, defense against ectoparasites, and prey capture [3,14]. Many venomous animals use their venom to capture prey, and indeed, amongst mammals, facilitating feeding is a major function of their venom [2]. It can be difficult, however, to observe predatory behaviors of a small, nocturnal, arboreal mammal under field conditions. Slow lorises also consume prey immediately rather than caching it for later consumption [14], such as shrews do [15]. Thus, the role of loris venom in prey capture remains unclear.

Like any efficient predator, slow lorises likely assess the noxiousness, escape potential, or other characteristics of prey species and alter their behavior accordingly, including the possible use of venom. Venoms are a metabolically expensive resource [16], and loris venom, which includes a rich diversity of compounds [1,17], is likely no exception. Spiders (*Cupiennius salei*) perform “venom metering” or selectively use venom depending upon prey characteristics [18]. Scorpions (*Parabuthus transvaalicus*) also adjust the amount of venom they inject, as well as its chemical composition, based on the intensity of threats they receive [19]. Changes in slow loris behavior depending on prey type may, thus, provide experimental evidence of venom use.

We collected detailed observations of slow loris predatory behavior to assess the hypothesis that slow lorises envenomate their prey. We used a combination of live observations and high-definition video recording to analyze the behavior of slow lorises capturing arthropods. We predicted that if slow lorises used venom to capture prey, they would perform pre-capture behaviors that served to arm them with BGE. Other behavioral signs of venom use would include release and recapture of prey, bite delivery and location, and, potentially, moderation of venom-related behaviors in response to prey size, escape potential, or toxicity. This study represents one of the first attempts to provide empirical evidence for the functional role of a unique mammalian trait, the toxic bite of the slow loris.

## 2. Materials and Methods

### 2.1. Slow Loris Subjects and Housing

Our subjects were *n* = 22 (11 male and 11 female) adult greater slow lorises (*Nycticebus coucang*) housed at Cikananga Wildlife Center (Pusat Penyelamatan Satwa Cikananga: PPSC) in West Java, Indonesia. These individuals were illegally wild-caught in Sumatra and were received by PPSC in October 2013 following their confiscation from a wildlife trader.

The slow lorises were housed indoors on a natural light regimen as solitary individuals (seven males and one female), mother-infant pairs (four females with their infants), or same-sex adult pairs (four males in two pairs and six females in three pairs). Groups were housed in similarly furnished wire-mesh cages measuring between 0.7 and 2.8 m^3^. Each loris was fed 60–150 g fruit/vegetable mix daily with ad libitum water. The slow lorises were also fed daily 5–6 crickets or mealworms in an enrichment device consisting of a garden pot full of leaf litter, which they had access to during experimental trials.

### 2.2. Prey Items

We experimentally presented the slow lorises with arthropods (*n* = 75) collected from the grounds of PPSC. We tested arthropods from seven orders, as well as millipedes (Class Diplopoda) for which we were unable to assign taxonomic order (Table 1). Following Nekaris [20], we operationally defined small prey as items easily covered by the loris’ hand, medium prey as overlapping the hand, and large items as several orders of magnitude larger than the hand. We defined escape potential as high for flying insects of the orders Hemiptera, Lepidoptera (adults), and Odonata, as well as the jumping insects of the orders Mantodea and Orthoptera [21], and as low for all other arthropods.

Because the cages were made of wire mesh with large gaps, arthropods could easily escape before being captured by a slow loris. To facilitate filming, we depressed locomotor behavior in the arthropods by exposing them to 15–20 min of cold (by placing them in a portable cooler), shaking them in a jar, or both treatments. We chose this approach to stimulate natural prey capture behavior without exposing the slow lorises to risks from glue or wire restraints.

### 2.3. Behavioral Experiments

We conducted 75 experimental tests of slow loris prey capture between February and April 2014. We conducted all behavioral tests in the slow lorises’ home cages to minimize disruption to the animals. We tested socially housed animals together with a single prey item for the pair. We did not want to withhold food from the slow lorises, so we were unable to control for motivation (hunger); however, we only completed one test per animal on a night of filming. Each individual received several practice trials to habituate them to being filmed.

For each trial, we presented the slow lorises with a prey item on a small arena (their plastic food dish inverted on the cage floor). The first author collected all-occurrences of behaviors (Table 2) from the start of the trial until prey capture and timed the trial [22]. Trials were filmed in high definition (25 frames per second) using a Canon EOS 7D SLR (Canon UK Ltd., Uxbridge, UK) and supplemental red light. After the slow loris finished consuming the prey item, all-occurrence behaviors were recorded for an additional 5 min. We suspended trials after 15 min if the slow loris did not capture the arthropod.

We used videos of prey strikes to record additional behavioral variables coded in duplicate using iMovie (Apple Inc., Cupertino, CA, USA). Because enclosure sizes varied, we scored the latency from entering the strike zone (within grabbing distance of the prey item) to prey capture. Other timed variables included total trial length, latency between capture and consumption, and consumption duration. Finally, we recorded whether the hand or mouth was used for capture, the location where the arthropod was initially bitten, the number of chewing cycles during consumption, and which parts of the arthropods were discarded.

### 2.4. Data Analysis

We compared attack latencies and rates of pre- and post-strike behaviors in relation to slow loris sex, social group, prey size, prey escape potential, prey noxiousness, and prey type (by order) using a multivariate general linear model (GLM). For pre-capture behaviors, we used all trials (*n* = 93 adult slow lorises with 75 prey items) in comparisons, while we used only data from the slow lorises that consumed prey items (60 of 75 trials) to analyze timed and post-capture behaviors. The infants housed with their mothers were not included in these totals or considered in data analysis. We tested for homoscedasticity in the GLM using Levene’s Test of Equality of Error Variances. Although most variables of interest were normally distributed, we adjusted degrees of freedom using Hotelling’s Trace. We compared rates of behaviors pre- and post-consumption using paired *t*-tests. We conducted all analyses in SPSS v. 21 (IBM Corporation, Armonk, NY, USA).

## 3. Results

### 3.1. The Prey Capture Sequence

The slow lorises eagerly approached prey items after they were introduced, and the capture sequence varied little for different arthropods consumed. In every trial, the slow loris visually targeted the arthropod, fixing its gaze on the prey item while quietly and deliberately approaching the arena. They rarely paused in their approach to perform intervening behaviors and struck quickly once within grasping distance of the prey. The slow lorises caught the arthropods using their hands in all but one trial. They performed bimanual grabs in ten captures (*n* = 64, 15.6%), left-handed grabs in 24 captures (37.5%), and right-handed grabs in 29 captures (45.3%). As a group, the slow lorises did not show a significant preference for the right or left hand (Chi-square test: X^2^ = 0.5, *p* = 0.5).

**Figure 1:**
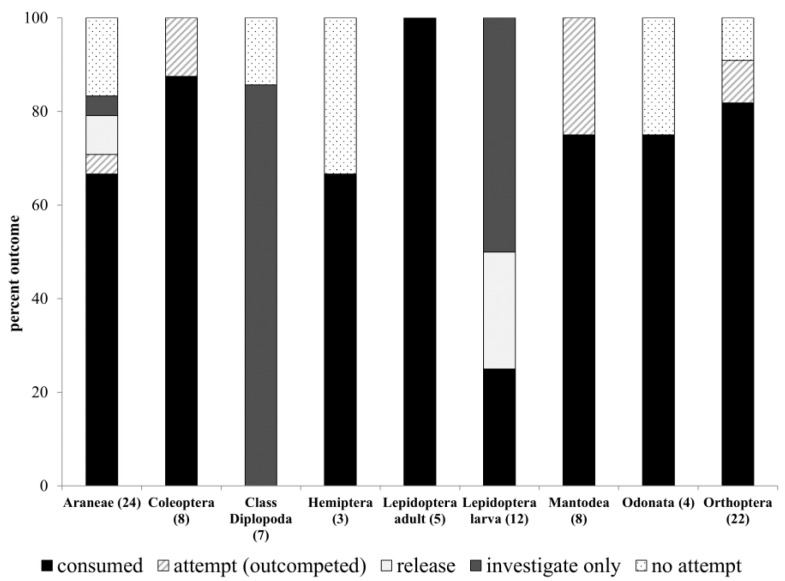
Outcomes of prey capture trials by arthropod order. A total of 75 trials were conducted, 18 with two adult slow lorises present, for a total of 93 capture opportunities (number per order is indicated on the x-axis). For attempt (outcompeted) trials, both slow lorises pursued the prey item.

The slow lorises readily consumed most types of prey (Figure 1). The only types of arthropods they captured and released or investigated without capture were caterpillars, millipedes, and spiders. The fatal strike was almost invariably delivered by decapitation, and slow lorises would often pause to reorient the prey item in their hands to bite off the head. Some smaller arthropods, including spiders and caterpillars, were placed entirely in the mouth prior to the initial bite. Most arthropods were consumed in their entirety, but sometimes, the slow lorises used the tongue to spit out wings from flying insects or discarded thicker portions of the exoskeleton from large beetles.

### 3.2. Venom-Related Behaviors

**Figure 2:**
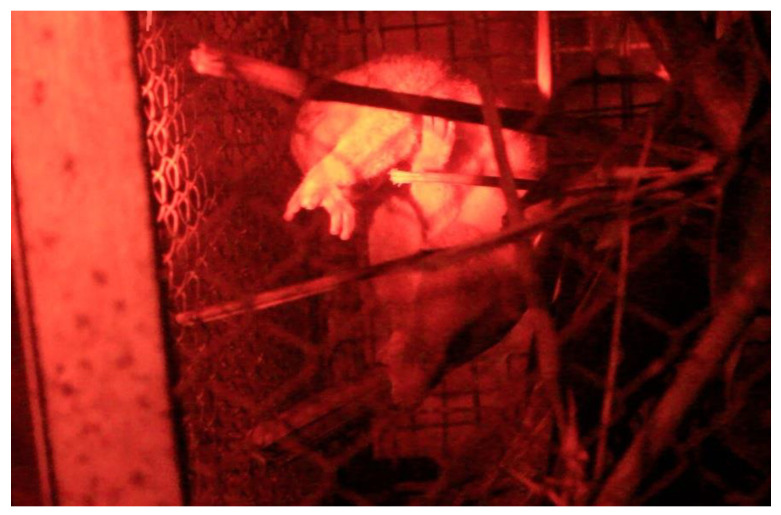
Male slow loris licks brachial gland during a trial with a larva of Lasiocampidae.

We only observed a slow loris lick its brachial gland on five occasions and only once outside the context of a larger grooming bout. In this case, (Figure 2), a male slow loris approached a caterpillar (Family Lasiocampidae) repeatedly, each time tongue-flicking rapidly. Without attempting to capture the caterpillar, he retreated to an elevated perch, raised his arm, and licked only the brachial gland without grooming anywhere else. Although he approached the caterpillar once more, he made no attempt to capture it. He did not assume the characteristic slow loris defensive posture, in which the arms are raised above the head [3], nor did he anoint himself with BGE. In fact, we never observed either of these behaviors in any individual outside of being caught by veterinarians for medical inspections. The other four events of brachial licking occurred as part of grooming bouts during trials with a spider (*Nephila pilipes*), a beetle (*Xystrocera festiva*), and two millipedes. Only the beetle was captured and consumed.

### 3.3. Effects of Prey Attributes

The multivariate model showed no main effects of sex or social condition or significant interactions between these variables with the fixed factors prey size, escape potential, toxicity, or prey order. Therefore, we did not control for sex or social condition in further analyses.

There was no main effect of prey size on pre-capture all-occurrence behaviors, but the comparison for behaviors surrounding consumption (post-capture all-occurrence behaviors and timed variables) was highly significant (MANOVA: Hotelling’s Trace = 2.3, F_24,82_ = 3.9, *p* < 0.001). Univariate tests revealed a longer mean consumption duration (308.9 ± 97.6 (SE) sec for large arthropods compared to 62.9 ± 10.4 s for medium and 38.8 ± 6.8 s for small arthropods; F_2,53_ = 12.4, *p* < 0.001), greater mean number of chewing cycles (381.3 ± 90.4 cycles for large arthropods compared to 120.8 ± 21.0 cycles for medium and 68.6 ± 13.3 cycles for small arthropods; F_2,53_ = 12.2, *p* < 0.001), and higher rate of grooming the hands following consumption of larger prey compared to other sizes (0.4 ± 0.1 licks/min for large arthropods compared to rates of 0.1 ± 0.03 for medium and 0.08 ± 0.06 for small arthropods; F_2,53_ = 12.4, *p* < 0.001).

The multivariate model was nonsignificant for pre-capture all-occurrence behaviors compared using escape potential as a fixed factor. There was a significant main effect of escape potential on timed behaviors (MANOVA: Hotelling’s Trace = 0.7, F_12,43_ = 2.7, *p* = 0.009). The slow lorises moved more quickly to strike prey with a greater escape potential, but this trend only neared significance (5.4 ± 1.6 s for high escape potential and 11.5 ± 3.0 s for low; F_1,54_ = 3.8, *p* = 0.06). The latency between capture and the first bite was longer for arthropods with higher escape potential (6.1 ± 1.3 s for high escape potential and 2.3 ± 0.5 s for low; F_1,54_ = 6.1, *p* = 0.02). All the arthropods with high escape potential were preferred food items, and none of the slow lorises ever refused to capture or consume any flying or jumping insects.

### 3.4. Effects of Prey Type

**Figure 3:**
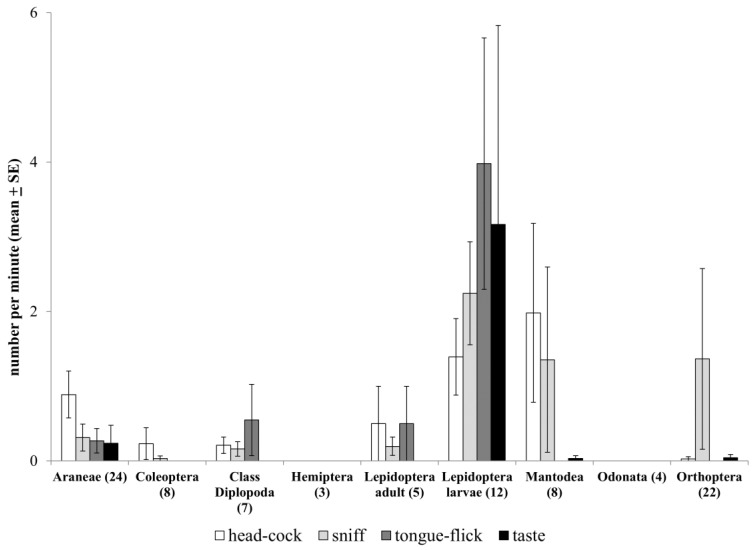
Pre-capture investigative behaviors (mean ± SE number per min) by arthropod order for all trial outcomes. A total of 75 trials were conducted, 18 with two adult slow lorises present, for a total of 93 capture opportunities (number per order indicated in parentheses on the x-axis). Slow lorises visually targeted the prey item during every capture attempt; therefore, this behavior is not depicted here.

The behavior of the slow lorises differed most dramatically in relation to the type of arthropod presented (Figure 3). The slow lorises never consumed millipedes, and only two of ten slow lorises presented with caterpillars consumed them (Figure 1). There was a significant main effect of prey order on pre-capture (Figure 3; MANOVA: Hotelling’s Trace = 1.3, F_72,594_ = 1.4, *p* = 0.03) and post-capture (MANOVA: Hotelling’s Trace = 3.9, F_84,247_ = 1.7, *p* = 0.002) all-occurrence behaviors.

The slow lorises mainly performed tongue-flicking behavior when confronted with Lepidoptera and millipedes and, occasionally, in response to spiders (Figure 3; F_9,83_ = 3.6, *p* = 0.001). Tongue-flicking lorises rapidly protruded their tongue in and out of their mouths while visually focusing on the arthropod (Supplemental Video S1). The slow lorises only tongue-flicked when they were close to the arena (within 0.5 m), and often, they tongue-flicked very near the prey item, almost but not quite touching it. The slow lorises tongue-flicked significantly more before than after prey-capture (mean of 0.4 ± 0.2 tongue-flicks/min before and 0.0 ± 0.0 flicks/min after; paired *t*-test: t_59_ = 2.0, *p* = 0.045), and we never observed this behavior post-consumption.

The slow lorises were also more likely to taste (directly lick) potentially toxic arthropods prior to catching them. Tasting and sniffing often occurred in conjunction with tongue-flicking but did not vary with prey order at statistically significant rates (Figure 3; taste: F_9,83_ = 0.9, *p* = 0.5; sniff: F_9,83_ = 0.6, *p* = 0.8). The slow lorises were more likely to approach and retreat multiple times when presented with caterpillars and millipedes than other prey types (F_9,83_ = 4.2, *p* < 0.001). Head-cocking occurred significantly more prior to capturing prey than after consumption (mean of 0.6 ± 0.2 times/min before and 0.02 ± 0.01 times/min after; paired *t*-test: t_59_ = 3.6, *p* = 0.001).

The slow lorises were slower to capture potentially noxious prey compared to more palatable items. The mean latency to strike was longer for noxious arthropods (13.1 ± 3.9 s for high toxicity and 5.7 ± 1.3 s for low; F_1,54_ = 4.1, *p* = 0.047) due to extremely long mean strike latencies for Lepidoptera larvae (31.7 ± 8.8 s; F_7,48_ = 3.5, *p* = 0.004). When toxic arthropods were captured, the mean latency to consume them was shorter compared to more palatable prey, although this difference only approached significance (2.5 ± 0.7 s for high toxicity and 5.4 ± 1.1 s for low; F_1,54_ = 3.7, *p* = 0.058).

Higher mean rates of licking the hands after consumption of potentially toxic prey also approached significance (0.2 ± 0.1 times/min for high toxicity and 0.1 ± 0.03 times/min for low; F_1,54_ = 3.5, *p* = 0.06). On several occasions, the observer saw a slow loris pause in approaching a noxious prey item, stopping to lick its hands carefully, and then resume the approach. One female completed this sequence of behavior three times in a single trial with a millipede, although she ultimately retreated after each approach without any capture attempts. However, pre-capture rates of hand-licking did not vary significantly with prey order, and the slow lorises licked their hands at higher rates after consuming prey than before capturing it (mean of 0.02 ± 0.01 times/min before and 0.15 ± 0.03 times/min after; paired *t*-test: t_59_ = −3.6, *p* = 0.001).

## 4. Discussion

### 4.1. Evidence for Envenomation of Prey

Overall, the slow lorises were eager to consume arthropod prey, confirming other studies that have shown that feeding arthropods can be enriching for slow lorises living in sanctuaries and rescue centers [23]. When capturing arthropod prey, the slow lorises visually targeted the prey, then, moved quickly and efficiently to subdue it without pausing to perform intervening behaviors, such as licking the brachial gland. The slow lorises did not release and recapture prey as if they were waiting for venom to take effect before delivering a fatal strike [24]. Instead, the slow lorises quickly decapitated or consumed the prey whole after capture. Similarly, wild slow lorises are also typically observed consuming arthropods whole [8], although the smaller pygmy loris has also been seen consuming the heads of arthropods first [10]. Taken together, these results do not support the prey capture hypothesis for loris venom. When the slow lorises did lick the brachial gland in this study, they did so in response to potentially toxic prey, most of which they did not attempt to capture. Therefore, it is possible that we observed this behavior being used in a defensive rather than predatory context.

We cannot discount the possibility that the methods we used to depress the locomotor activity of the prey items we tested could have altered the capture behavior of the slow lorises by rendering these prey items easier to capture, making the use of venom unnecessary. Yet, the similarity between prey capture behaviors we observed by these captive slow lorises and those observed in wild slow lorises [8,10] suggests this was not the case. Given the relatively short time that these slow lorises had been in captivity, it also seems likely that they would exhibit predatory behaviors more similar to those of wild conspecifics than slow lorises which had been born in captivity or resided longer in human care.

This study adds to the growing body of evidence that slow lorises use their venom for intraspecific competition [12]. However, we did not observe any venom-related behaviors related to competition for prey when we conducted trials with pair-housed slow lorises, even though we only provided pairs with a single prey item in each trial. We only observed agonistic behavior during one trial when a pair of unrelated adult females actively fought over a mantis (*Tenodera aridifolia*). The female who captured the mantis threatened and bit the second female when she attempted to grab it; however, she did not lick the brachial gland prior to biting her conspecific. We never saw agonistic behavior between mothers and their infants, which is not surprising given that slow lorises are known to co-feed extensively in family groups before reaching dispersal age [25]. All the individuals in this study were provisioned, so they also may have been less motivated to fight over prey than wild slow lorises. Thus, it is unclear from this study whether competition over prey is a context in which slow lorises envenomate one another. However, sex-specific patterns in venom-related wounds observed in wild slow lorises suggest that envenomation usually occurs in disputes over territory or mates [12].

We observed slow lorises carefully licking their fingers and palms prior to investigating toxic prey. This behavior was similar to observations that male slow lorises anoint themselves prior to agonistic encounters by grooming their brachial gland along with their hair [3]. However, in this case, the behavior only involved salivary secretions, and we did not observe grooming of the brachial gland. This raises an interesting question: is it possible that there is a substance in the saliva itself that protects against chemically defended prey? Other animals secrete protective chemicals into the oral cavity; for example, horned lizards (*Phrynosoma* spp.) produce a pharyngeal mucous that incapacitates stinging ants during swallowing [26]. Experimentally, exposure to slow loris BGE is incapacitating or fatal to small arthropods (maggots and spiders), suggesting it could play a role in repelling ectoparasites like ticks [27]. Furthermore, the parotid and submandibular glands of *N. coucang* contain unusually large secretory granules of unknown function [28], which contain large amounts of kallikrein, toxins also present in the saliva of venomous shrews and solenodons. The saliva of Javan slow lorises (*N. javanicus*) also is dominated by a 25 kDa protein, identified as a complement component 1r (C1R). C1rs are known to cause inflammation and swelling [1]. Given these observations and the entirely unique “two-step” nature of their venom system, in which saliva “activates” or “charges” the slow loris venom [1,17], perhaps further investigation of the chemical constituents of the saliva will reveal novel functions complementary to the BGE. Such functionality is not unexpected given that the venom glands for most venomous mammals are modified submaxillary salivary glands [14].

### 4.2. Effects of Prey Attributes on Loris Predatory Behavior

Due to the difficulties of observing slow lorises in the wild, field researchers studying food consumption are typically unable to identify the type of arthropods being consumed [8,11]. These captive experiments thus generated novel information about slow loris preferences and behavior based on arthropod type. Based on prey size and escape potential, slow lorises showed some predictable differences in the timing of prey capture but not pre-capture behavior. Slow lorises took longer to consume larger but not more toxic prey [29], similar to how wild pygmy lorises spend more time feeding on larger arthropods [10]. They were faster to grab arthropods with higher escape potential but slower to consume them following the strike. It is possible that slow lorises took care in re-orienting these preferred food items for decapitation, avoiding a potential escape between the initial capture and the fatal bite. Compared to benign arthropods, slow lorises also started consuming potentially toxic prey more quickly following capture, perhaps to avoid injury by defended prey [30].

Although we observed few effects of arthropod size on capture behavior, slow lorises are known to consume birds and other larger vertebrates under free-ranging conditions [3,9], and it is possible that all the arthropods we tested were small in comparison. Thus, additional observations of slow loris predatory behavior with other vertebrates may be necessary to dismiss the prey capture hypothesis for slow loris venom conclusively. However, Streicher et al. [10] observed captive slow lorises capture and consume avian prey (yellow-vented bulbuls, *Pycnonotus goiavier*) and did not report any venom-related behaviors; rather, the birds were dispatched by a lunge and manual grab, followed by a bite to the neck. Yet, injecting mice with a mixture of slow loris venom extract and saliva was fatal in another experiment [4], again suggesting the use of venom to subdue larger prey is a possibility. Since slow lorises do use their venom against other slow lorises, the ability of their venom to subdue larger mammalian prey may be a side effect of the use of venom for intraspecific competition [12].

Additionally, we cannot discount any potential influences of dental condition on the captive slow lorises involved in this study. Wildlife traders often remove teeth from slow lorises as protection from their venomous bite [31]; although, the animals in this study had not been subjected to this practice. Yet, dental disease was the second most common source of morbidity (following external wounds) in this study group during their first six months in the rescue center, when these experiments were performed [32]. Although we only performed trials on animals that were apparently healthy and regularly consumed their routine diets, we cannot conclusively determine whether their dental condition could have affected their behavior in this study, particularly since the toothcomb, which is sensitive to dental infection, is known to provide a pathway for venom transmission [4].

### 4.3. Sensory Modalities Used for Prey Assessment

The most striking differences in pre-capture behavior occurred as a function of prey type. During their lengthier approaches towards noxious prey, slow lorises performed a suite of behaviors including sniffing, directly licking prey, and tongue-flicking that may indicate they are using chemosensory information to assess prey toxicity or as a defensive display to advertise their own toxicity [33].

Slow lorises in this study selectively tongue-flicked in response to noxious arthropods that they did not attempt to capture, suggesting the behavior could have served as a warning signal. It has been suggested that slow loris venom is part of a suite of aposematic signals, including serpentine locomotion, dorsal striping, facial masks, and snake-like vocalizations, that may be part of a multimodal signaling complex used to mimic cobras (*Naja* spp.) [3,34]. Younger and more aggressive slow lorises show greater color contrast in both their facial masks [34] and dorsal stripes [35] compared to others. These contrasting features may be an honest advertisement of aggressive potential in younger slow lorises, which often engage in aggressive intraspecific encounters during dispersal at this age [35]. Slender lorises also perform snake-like defensive displays that involve spitting and positioning the hands to mimic a cobra’s hood [36] but have not been observed to tongue-flick during these displays. However, slow lorises have not been observed tongue-flicking at predators, and the behavior may instead (or also) serve a chemosensory function.

Chemosensation has been largely understudied in nonhuman primates [37], but like other strepsirrhines, slow lorises have a well-innervated vomeronasal organ with a rich array of sensory receptors that likely play a role in olfactory discrimination as well as taste [37,38]. Unlike other mammals, nonhuman primates do not seem to perform classic flehmen behaviors. This behavior has, however, been observed frequently in Javan slow lorises (Nekaris, K.A.I., personal communication), and mouse lemurs (*Microcebus* spp.) reportedly engage in a reflexive tongue-lapping behavior in response to urine administration [37]. Tongue-flicking has also been described in common marmosets (*Callithrix jacchus*) as part of a sequence of behaviors performed to investigate scent marks [37], occurring in a sociosexual context [39]. Similarly, there is indirect evidence that captive pygmy lorises use chemoreception to evaluate potential mates [40,41], although they investigated scent marks by sniffing and licking, not tongue-flicking. However, no study has reported tongue-flicking by slow lorises occurring in a predatory context.

The function of tongue-flicking in squamates is to deliver complex volatile chemicals to the vomeronasal organ [42]. Forked tongues enable snakes to track prey by means of chemical tropotaxis, in which simultaneous input received from paired sensors on each tongue encodes information about prey location [43]. Interestingly, slow lorises also have a dual tongue structure, which is thought to have the primary function of cleaning the toothcomb. Pygmy lorises (*X. pygmaeus*) missing the sublingua are able to maintain adequate dental health, raising the possibility that the second tongue may serve other functions [44]. Perhaps slow lorises also use this paired structure to encode information about the location of chemically defended prey.

An alternative explanation for tongue-flicking is that it serves as a displacement behavior, which is a typically self-directed behavior that appears irrelevant to the circumstances in which they are performed and is associated with anxiety or stress [45]. Chertoff et al. [46] observed repeated tongue-flicking in a single Balinese long-tailed macaque (*Macaca fascicularis*) and concluded it represented an anxiety-related displacement behavior due to its idiosyncratic appearance and temporal relationship with agonistic interactions. This behavior was much more widespread in our study sample (eight individuals tongue-flicked one or more times during experiments), and the observation that this behavior was selectively aimed towards noxious prey suggests that tongue-flicking was purposeful in this context.

Although slow lorises displayed interesting chemosensory behaviors, the dominant sense used to identify and capture prey in this study was clearly vision. Like slender lorises [20], the slow lorises also frequently moved their heads from side to side to side on a horizontal axis while they gazed at prey items. This ‘head-cocking’ behavior likely aids in localizing objects in space [47]. The first primates are thought to have been small, nocturnal insectivores much like slow lorises. In this scenario, the prehensile grasping motion used to capture prey demonstrated by the slow lorises here would have been the driving force for the evolution of ocular convergence, grasping hands, and other characteristics that exemplify the primate order [20,48]. Like other predators, slow lorises are clearly using multimodal input to assess prey characteristics and adjusting their behavior as new information becomes available at the limits of these sensory inputs [49].

## 5. Conclusions

We found little evidence to support the hypothesis that greater slow lorises use their venom to subdue arthropod prey. We did observe slow lorises performing unique behaviors such as tongue-flicking that indicate they are using chemosensory information to assess other animals. However, slow lorises are skilled at manually capturing small prey, and our data suggest they do not rely on venom for this purpose. This conclusion is consistent with other studies examining the evolutionary ecology of slow loris venom, which have found that venom is typically used in the context of intraspecific competition [12], rather than in predator-prey interactions.

## Figures and Tables

**Table 1:** Arthropods used to test prey capture behavior by 22 adult great slow lorises at PPSC, Indonesia.

Order: Family	Species	*N*
Araneae	Total	19
Araneidae	*Argiope* sp.	3
Araneidae	unknown sp.	7
Nephilidae	*Nephila pilipes*	6
Sparassidae	unknown sp.	3
Coleoptera	Total	7
Cerambycidae	*Xystrocera festiva*	4
Scarabaeidae	*Phyllophaga* sp.	1
Scarabaeidae	*Xylotrupes* gideon	1
Scarabaeidae	*Xylotrupes* sp.	1
Class Diplopoda (order unknown)	Total unknown sp.	5
Hemiptera	Total	2
Cicadidae	*Dundubia vaginata*	2
Lepidoptera (adults)	Total	5
Noctuidae	unknown sp.	1
Nymphalidae	*Charaxes nobilis*	2
Nymphalidae	*Junonia* sp.	1
Pyralidae	unknown sp.	1
Lepidoptera (larvae)	Total	10
Arctiidae	unknown sp.	2
Lasiocampidae	unknown sp.	3
Notodontidae	unknown sp.	2
Nymphalidae	unknown sp.	3
Mantodea	Total	6
Mantidae	*Amantis* sp.	1
Mantidae	*Hierodula* sp.	1
Mantidae	*Hierodula vitrea*	1
Mantidae	*Tenodera aridifolia*	3
Odonata	Total	3
Libellulidae	*Orthetrum sabina*	2
Libellulidae	*Pantala flavescens*	1
Orthoptera	Total	18
Acrididae	*Valanga nigricornis*	3
Acrididae	*Oxya chinensis*	6
Gryllacrididae	*Gryllacris signifera*	1
Gryllidae	*Acheta domesticus*	1
Gryllidae	*Gryllus* sp.	1
Tettigonidae	*Conocephalus* sp.	1
Tettigonidae	*Holochlora* sp.	1
Tettigonidae	*Mecapoda* sp.	1
Tettigonidae	*Xiphidion* sp.	1
Tettigonidae	unknown sp.	2
Arthropods	Total	75

**Table 2:** Ethogram for greater slow loris behaviors pre and post prey capture.

Behavior	Operational Definition
Investigatory and Preparatory Behaviors	
scent-marking	Rubbing the face, chest, or perineum along substrates; or urine marking.
circling	Radial movement above the arena without descending to the cage floor, while fixing attention on prey.
brachial lick	Making contact between the tongue and the brachial gland.
lick hands	Using the tongue to apply saliva to the fingers or palms.
groom other	Licking or cleaning anywhere on the body except the brachial gland or hands using the tongue, toothcomb, or grooming claw.
approach and retreat	Moving away from proximity to the prey item after investigating it with no attempted capture.
grab and release	Letting go of the prey item after capture without attempting to consume the item.
Sensory Behaviors	
visually target	Fixing the gaze on the prey item and maintaining eye contact with it while moving toward capture.
head-cocking	Slowly turning the head on a 180-degree axis, while focusing visual attention on the prey item.
ear twitch	Visibly moving the ears or moving the head to orient them toward the prey.
sniff	Repeatedly moving the nose to rapidly inhale air or moving the nose along a substrate while inhaling.
tongue-flicking	Rapidly moving the tongue in and out of the mouth without contacting the prey item or another substrate.
taste (lick)	Moving the tongue in contact with the prey without attempting to consume it or using the teeth or lips.

## Data Availability

Complete data are available from the authors upon request.

## Supplementary Materials

The following supporting information can be downloaded at: https://www.mdpi.com/article/10.3390/ani14101438/s1, Video S1: Greater slow loris tongue-flicking.

## References

[1] Fitzpatrick L.L., Ligabue-Braun R., Nekaris K.A. (2023). Slowly Making Sense: A Review of the Two-Step Venom System within Slow (*Nycticebus* spp.) and Pygmy Lorises (*Xanthonycticebus* spp.). Toxins.

[2] Ligabue-Braun R., Verli H., Carlini C.R. (2012). Venomous mammals: A review. Toxicon.

[3] Nekaris K.A.I., Moore R.S., Rode J., Fry B.G. (2013). Mad, bad and dangerous to know: The biochemistry, ecology and evolution of slow loris venom. J. Venom. Anim. Toxins Incl. Trop. Dis..

[4] Alterman L., Alterman L., Doyle G., Izard M.K. (1995). Toxins and toothcombs: Potential allospecific chemical defenses in *Nycticebus* and *Perodicticus*. Creatures of the Dark.

[5] Wilde H. (1972). Anaphylactic shock following bite by a slow loris, *Nycticebus coucang*. Am. J. Trop. Med. Hyg..

[6] Fry B.G., Roelants K., Champagne D.E., Scheib H., Tyndall J.D., King G.F., Nevalainen T.J., Norman J.A., Lewis R.J., Norton R.S. (2009). The toxicogenomic multiverse: Convergent recruitment of proteins into animal venoms. Annu. Rev. Genom. Hum. Genet..

[7] Rode-Margono E.J., Rademaker M., Wirdateti, Strijkstra A., Nekaris K.A.I. (2015). Noxious arthropods as potential prey of the venomous Javan slow loris (*Nycticebus javanicus*) in a West Javan volcanic agricultural system. J. Nat. Hist..

[8] Cabana F., Dierenfeld E., Wirdateti W., Donati G., Nekaris K.A.I. (2017). The seasonal feeding ecology of the Javan slow loris (*Nycticebus javanicus*). Am. J. Phys. Anthropol..

[9] Starr C., Nekaris K.A.I. (2013). Obligate exudativory characterizes the diet of the pygmy slow loris *Nycticebus pygmaeus*. Am. J. Primatol..

[10] Streicher U., Wilson A., Collins R.L., Nekaris K.A.-I., Masters J., Gamba M., Génin F. (2013). Exudates and Animal Prey Characterize Slow Loris (*Nycticebus pygmaeus*, *N. coucang* and *N. javanicus*) Diet in Captivity and After Release into the Wild. Leaping Ahead: Advances in Prosimian Biology.

[11] Wiens F., Zitzmann A., Hussein N.A. (2006). Fast food for slow lorises: Is low metabolism related to secondary compounds in high-energy plant diet?. J. Mammal..

[12] Nekaris K., Campera M., Nijman V., Birot H., Rode-Margono E.J., Fry B.G., Weldon A., Wirdateti W., Imron M.A. (2020). Slow lorises use venom as a weapon in intraspecific competition. Curr. Biol..

[13] Schendel V., Rash L.D., Jenner R.A., Undheim E.A.B. (2019). The Diversity of Venom: The Importance of Behavior and Venom System Morphology in Understanding Its Ecology and Evolution. Toxins.

[14] Rode-Margono J.E., Nekaris K.A.-I. (2015). Cabinet of Curiosities: Venom Systems and Their Ecological Function in Mammals, with a Focus on Primates. Toxins.

[15] Pearson O.P. (1942). On the cause and nature of a poisonous action produced by the bite of a shrew (*Blarina brevicauda*). J. Mammal..

[16] Morgenstern D., King G.F. (2013). The venom optimization hypothesis revisited. Toxicon.

[17] Hagey L.R., Fry B.G., Fitch-Snyder H., Gursky S., Nekaris K.A.I. (2007). Talking defensively: A dual use for the brachial gland exudate of slow and pygmy lorises. Primate Anti-Predator Strategies.

[18] Wigger E., Kuhn-Nentwig L., Nentwig W. (2002). The venom optimisation hypothesis: A spider injects large venom quantities only into difficult prey types. Toxicon.

[19] Nisani Z., Hayes W.K. (2011). Defensive stinging by *Parabuthus transvaalicus* scorpions: Risk assessment and venom metering. Anim. Behav..

[20] Nekaris K.A.I. (2005). Foraging behaviour of the slender loris (*Loris lydekkerianus lydekkerianus*): Implications for theories of primate origins. J. Hum. Evol..

[21] Schiel N., Souto A., Huber L., Bezerra B.M. (2010). Hunting strategies in wild common marmosets are prey and age dependent. Am. J. Primatol..

[22] Altmann J. (1974). Observational Study of Behavior: Sampling Methods. Behaviour.

[23] Chatpongcharoen P., Campera M., Laithong P., Gibson N.L., Nekaris K. (2021). Naturalising diet to reduce stereotypic behaviours in slow lorises rescued from wildlife trade. Appl. Anim. Behav. Sci..

[24] Cooper W.E. (2003). Foraging mode and evolution of strike-induced chemosensory searching in lizards. J. Chem. Ecol..

[25] Maynard K.Q., Birot H., Campera M., Imron M.A., Jasso del Toro C., Poindexter S.A., Nekaris K.A.I. (2021). Slow learning of feeding skills in a nocturnal extractive forager. Anim. Behav..

[26] Sherbrooke W.C., Schwenk K. (2008). Horned lizards (*Phrynosoma*) incapacitate dangerous ant prey with mucus. J. Exp. Zool. Part A-Ecol. Genet. Physiol..

[27] Grow N.B., Wirdateti, Nekaris K.A.I. (2015). Does toxic defence in *Nycticebus* spp. relate to ectoparasites? The lethal effects of slow loris venom on arthropods. Toxicon.

[28] Tandler B., Pinkstaff C.A., Nagato T., Phillips C.J. (1996). Giant secretory granules in the ducts of the parotid and submandibular glands of the slow loris. Tissue Cell.

[29] Lappin A.K., German M. (2005). Feeding behavior modulation in the leopard lizard (*Gambelia wislizenii*): Effects of noxious versus innocuous prey. Zoology.

[30] Kornilev Y.V., Natchev N.D., Lillywhite H.B. (2023). Perils of ingesting harmful prey by advanced snakes. Biol. Rev..

[31] Nijman V., Spaan D., Rode-Margono E.J., Wirdateti, Nekaris K.A.I. (2017). Changes in the primate trade in Indonesian wildlife markets over a 25-year period: Fewer apes and langurs, more macaques, and slow lorises. Am. J. Primatol..

[32] Fuller G., Eggen W.F., Wirdateti W., Nekaris K.A.I. (2018). Welfare impacts of the illegal wildlife trade in a cohort of confiscated greater slow lorises, *Nycticebus coucang*. J. Appl. Anim. Welf. Sci..

[33] Glendinning J.I. (2007). How Do Predators Cope with Chemically Defended Foods?. Biol. Bull..

[34] Nekaris K.A.-I., Weldon A., Imron M.A., Maynard K.Q., Nijman V., Poindexter S.A., Morcatty T.Q. (2019). Venom in furs: Facial masks as aposematic signals in a venomous mammal. Toxins.

[35] Nekaris K.A.I., Campera M., Watkins A.R., Weldon A.V., Hedger K., Morcatty T.Q. (2021). Aposematic signaling and seasonal variation in dorsal pelage in a venomous mammal. Ecol. Evol..

[36] Nekaris K.A.I., Pimley E.R., Ablard K.M., Gursky S., Nekaris K.A.I. (2007). Predator defense by slender lorises and pottos. Primate Anti-Predator Strategies.

[37] Evans C.S. (2006). Accessory chemosignaling mechanisms in primates. Am. J. Primatol..

[38] Loo S.K., Kanagasu R. (1972). Vomeronasal organ in tree shrew and slow loris. J. Anat..

[39] Kendrick K.M., Dixson A.F. (1984). A quantitative description of copulatory and associated behaviors of captive marmosets (*Callithrix jacchus*). Int. J. Primatol..

[40] Fisher H.S., Swaisgood R.R., Fitch-Snyder H. (2003). Odor familiarity and female preferences for males in a threatened primate, the pygmy loris *Nycticebus pygmaeus*: Applications for genetic management of small populations. Naturwissenschaften.

[41] Fisher H.S., Swaisgood R.R., Fitch-Snyder H. (2003). Countermarking by male pygmy lorises (*Nycticebus pygmaeus*): Do females use odor cues to select mates with high competitive ability?. Behav. Ecol. Sociobiol..

[42] Martinez-Marcos A., Ubeda-Banon I., Halpern M. (2001). Neural substrates for tongue-flicking behavior in snakes. J. Comp. Neurol..

[43] Schwenk K. (1994). Why snakes have forked tongues. Science.

[44] Nadler T., Schwierz E., Streicher U. (2013). Pygmy lorises (*Nycticebus pygmaeus*) without sublingua. Vietnam. J. Primatol..

[45] Maestripieri D., Schino G., Aureli F., Troisi A. (1992). A modest proposal: Displacement activities as an indicator of emotions in primates. Anim. Behav..

[46] Chertoff S., Wandia I.N., Leca J.-B. (2023). Tongue-flicking: An idiosyncratic displacement behaviour in a free-ranging and urban-dwelling population of Balinese long-tailed macaques. Behaviour.

[47] Pariente G., Doyle G.A., Martin R.D. (1979). The role of vision in prosimian behavior. The Study of Prosimian Behavior.

[48] Cartmill M. (1992). New views on primate origins. Evol. Anthropol..

[49] Page R.A., Schnelle T., Kalko E.K.V., Bunge T., Bernal X.E. (2012). Sequential assessment of prey through the use of multiple sensory cues by an eavesdropping bat. Naturwissenschaften.

